# Successful Reconstruction of a Physiological Circuit with Known Connectivity from Spiking Activity Alone

**DOI:** 10.1371/journal.pcbi.1003138

**Published:** 2013-07-11

**Authors:** Felipe Gerhard, Tilman Kispersky, Gabrielle J. Gutierrez, Eve Marder, Mark Kramer, Uri Eden

**Affiliations:** 1 Brain Mind Institute, Ecole Polytechnique Fédérale de Lausanne (EPFL), Lausanne, Switzerland; 2 Biology Department and Volen Center, Brandeis University, Waltham, Massachusetts, United States of America; 3 Group for Neural Theory, École Normale Supérieure, Paris, France; 4 Department of Mathematics and Statistics, Boston University, Boston, Massachusetts, United States of America; Indiana University, United States of America

## Abstract

Identifying the structure and dynamics of synaptic interactions between neurons is the first step to understanding neural network dynamics. The presence of synaptic connections is traditionally inferred through the use of targeted stimulation and paired recordings or by post-hoc histology. More recently, causal network inference algorithms have been proposed to deduce connectivity directly from electrophysiological signals, such as extracellularly recorded spiking activity. Usually, these algorithms have not been validated on a neurophysiological data set for which the actual circuitry is known. Recent work has shown that traditional network inference algorithms based on linear models typically fail to identify the correct coupling of a small central pattern generating circuit in the stomatogastric ganglion of the crab *Cancer borealis*. In this work, we show that point process models of observed spike trains can guide inference of relative connectivity estimates that match the known physiological connectivity of the central pattern generator up to a choice of threshold. We elucidate the necessary steps to derive faithful connectivity estimates from a model that incorporates the spike train nature of the data. We then apply the model to measure changes in the effective connectivity pattern in response to two pharmacological interventions, which affect both intrinsic neural dynamics and synaptic transmission. Our results provide the first successful application of a network inference algorithm to a circuit for which the actual physiological synapses between neurons are known. The point process methodology presented here generalizes well to larger networks and can describe the statistics of neural populations. In general we show that advanced statistical models allow for the characterization of effective network structure, deciphering underlying network dynamics and estimating information-processing capabilities.

## Author Summary

To appreciate how neural circuits control behaviors, we must understand two things. First, how the neurons comprising the circuit are connected, and second, how neurons and their connections change after learning or in response to neuromodulators. Neuronal connectivity is difficult to determine experimentally, whereas neuronal activity can often be readily measured. We describe a statistical model to estimate circuit connectivity directly from measured activity patterns. We use the timing relationships between observed spikes to predict synaptic interactions between simultaneously observed neurons. The model estimate provides each predicted connection with a curve that represents how strongly, and at which temporal delays, one circuit element effectively influences another. These curves are analogous to synaptic interactions of the level of the membrane potential of biological neurons and share some of their features such as being inhibitory or excitatory. We test our method on recordings from the pyloric circuit in the crab stomatogastric ganglion, a small circuit whose connectivity is completely known beforehand, and find that the predicted circuit matches the biological one — a result other techniques failed to achieve. In addition, we show that drug manipulations impacting the circuit are revealed by this technique. These results illustrate the utility of our analysis approach for inferring connections from neural spiking activity.

## Introduction

Nervous systems show highly complex dynamics. This complexity originates from the intrinsic dynamics of each neuron, from its synaptic connections, and modulation state [1]–[3]. Unfortunately, information about synaptic relationships is generally sparse or often completely missing (see, e.g. [4]–[6], and references therein). Moreover, the inference of effective connectivity is based on limited information, such as the timing of spikes emitted by a subset of all neurons in the network. Here, effective connectivity is considered to be the network of directed, causal effects of one neural element over another (as opposed to structural or functional connectivity, see [7]). We can use spike trains to estimate effective connectivity networks, but how these effective networks relate to actual connectivity remains an open question [8]–[10].

There are many ways to build effective networks based on observed spiking activity. A commonly used network inference algorithm is Granger causality analysis [11], [12]. The strength of a causal link between two network nodes is measured by how well the knowledge of past activity of one node helps to predict the activity of the other node. Granger causality analysis has been applied to a variety of different imaging data at different spatial scales of brain activity [13]–[19], including spiking activity [20]–[23]. However, an inherent difficulty exists in validating these inference techniques because the underlying, true synaptic connectivity is typically not known. Usually, connectivity inference algorithms are validated on simulated data sets [15], [20], [24]–[28], and it remains largely unknown how well their predictions match the underlying structural connectivity.

In a recent study, Kispersky et al. [29] applied a linear Granger causality analysis to spiking activity from a physiological preparation, whose circuitry is well studied and understood [30], [31]. The analysis suggested an effective connectivity pattern of a three-node circuit that did not match the known physiological connectivity. The authors attributed this result to the presence of strong oscillatory components of the spiking activity and the inability of the analysis to capture the intrinsic pacemaker rhythm.

In this paper, we will continue the analysis of the spike train data with the goal of inferring network consistent with known connectivity. Generalized linear models take into account the point process nature of spike trains and have been used to infer connectivity in other biological neuronal networks [9], [23], [32]–[34]. Here, we will show for the first time that this approach, based on spike train data only, can identify relative connection strengths that match the known physiology of the pyloric circuit of the stomatogastric ganglion (STG) of the crab even though synaptic transmission in the pyloric circuit is graded and only partly mediated by spikes. If a threshold is applied on the estimated connection strengths, the physiological connectome of the circuit can be correctly reconstructed from the model.

To obtain this result, it is important to consider the functional shape and magnitude of the interactions in the model rather than statistical significance as it is classically quantified by Granger causality analysis. In the second part of the study, we show that both a nonlinear point process model and our measure of coupling strength are necessary to successfully infer the connectivity.

Finally, we show that inference using the point process model is robust to parameter changes, can be reproduced across several independent biological data sets, and can be used to predict how altered connectivity affects network function, i.e., the generation of the triphasic burst pattern. We demonstrate the ability of the method to track changes in the effective network connectivity structure caused by partial blocking of individual membrane currents or synaptic transmission. Our results add to the evidence in favor of applying point process statistical models to capture the statistics of spike trains. They constitute the first step toward the analysis of the relationship between structure and activity of larger neural circuits.

## Results

### A point process model and a direct measure of the coupling filter can correctly infer the known STG connectivity

**Figure 1:**
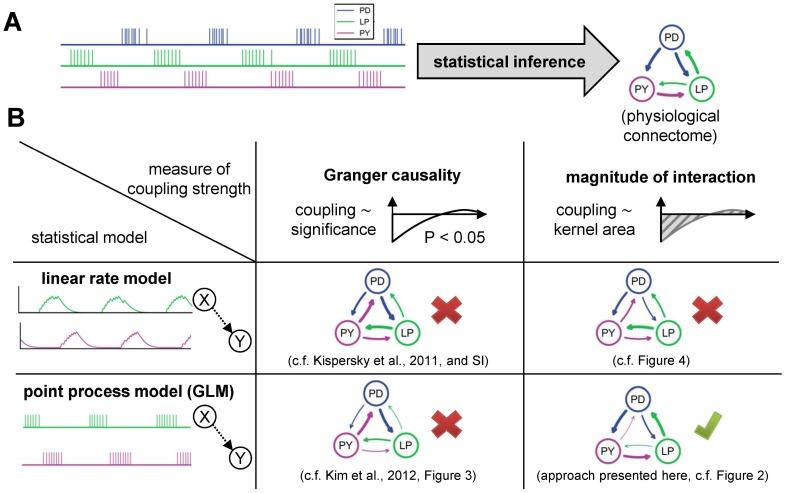
Inferring network connectivity of the pyloric circuit of the crab stomatogastric ganglion (STG) based on extracellular spike train recordings. **A**, Statistical models fitted on spike train activity (left) can be used to infer the effective coupling. The effective coupling should match the physiologically known diagram of the pyloric circuit (right). All synaptic couplings in the pyloric circuit are inhibitory. **B**, Comparison of algorithms for network inference. Neural activity can be either described in a firing rate model, e.g., in classical time series analysis, or using a point process model or generalized linear model (GLM). For both models, couplings are introduced as interaction kernels between the stochastic processes. The strength of the interaction can be either quantified through its statistical significance, i.e., a Granger causality-type measure, or through the magnitude of the interaction, as measured by the net area under the interaction kernel. Only the combination of a point-process-based generalized linear model with the definition of coupling strength as the magnitude of the interaction is able to recover a connectivity that is consistent with physiology (lower right). All other combinations of models and measures infer inaccurate connectivity patterns.

Extracellular recordings were obtained from three units of the crab stomatogastric ganglion (STG), which produce the pyloric rhythm [31]. Spike train activity follows a triphasic pattern starting with bursts of the anterior burster/pyloric dilator neurons (AB/PD, abbreviated as PD in the following), followed by sequential activation of the lateral pyloric neuron (LP) and pyloric neurons (PY) (Figure 1A, left). Neurons fired stereotypical bursts with a similar number of spikes within each burst over the whole recording session (mean Fano factor 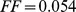, calculated as the variance over the mean of the distribution of spike counts per burst, averaged over the three neurons).

The physiological connections between the three units of the stomatogastric nervous system responsible for the pyloric rhythm are well understood [31] (Figure 1A, right). Notably, all synaptic connections are inhibitory, and there is no direct synaptic coupling from the PY neuron to the PD unit. Synapses can be qualitatively classified as weak and strong [35]–[38] (Figure 1A, right, strength indicated by line width).

A central question is: Given the spike trains, can we infer the connectivity of the circuit? Kispersky et al. demonstrated that in the presence of the strong oscillatory components, Granger causality analysis based on a linear firing rate model is unable to deduce the physiological connectivity pattern [29]. Instead, it identifies three strong interactions following the sequential activation of the PD, LP, and PY neurons (Figure 1B, upper left).

Our results show that two modifications to the approach of [29] permit accurate inference of the physiological circuit. First, the linear rate model is replaced by a nonlinear point process model that takes into account the structure of the data. Second, rather than basing the strength of the coupling on a statistical significance criterion as in Granger causality analysis [23], we propose to measure coupling strength directly as the magnitude of the estimated, directed coupling between two spike trains. With these two modifications, a statistical fit to the data can approximately recover the structure of the synaptic circuitry between the three units (Figure 1B, lower right; note that the missing physiological connection possesses the weakest coupling strength). Any other possible combination of model and coupling measure leads to inaccurate reconstructions (Figure 1B).

**Figure 2:**
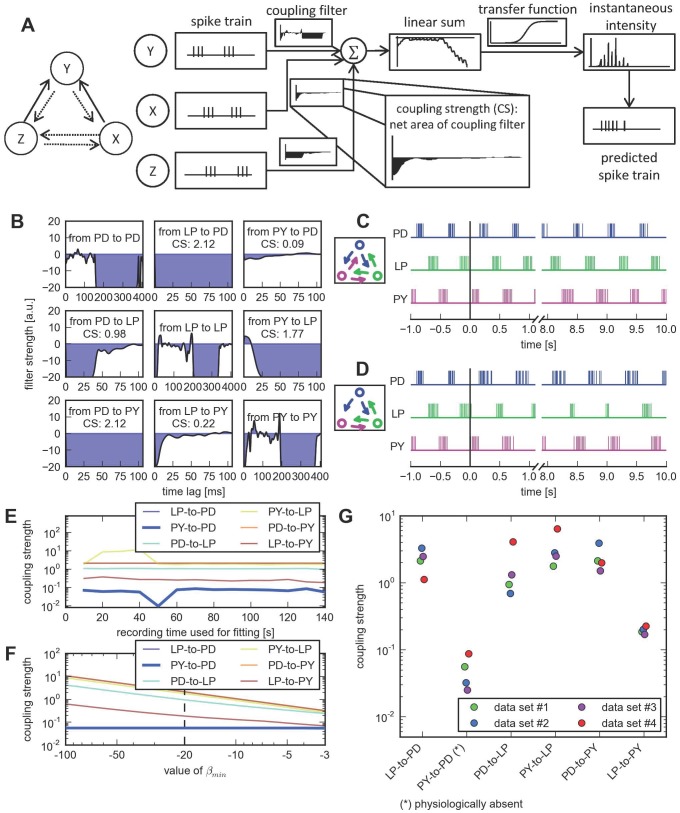
A point process model provides a good fit to the experimental data and recovers the known physiological connectivity. **A**, Schematic overview of the point process generalized linear model (GLM). One model is fit per neuron, conditioning its activity on its own previous activity (Y) and the activity of all other simultaneously recorded neurons (X, Z). Spike trains are convolved with filters conceptually similar to spike-triggered currents. All contributions are linearly summed and passed through a static sigmoidal nonlinearity. Spikes are assumed to be a sample from the instantaneous intensity function. Coupling strength between two neurons is defined as the net area under the coupling filter. **B**, Maximum-likelihood filters. Filters indicate how much the firing activity of the postsynaptic neuron is modulated by a spike in the presynaptic neuron at a specified lag. Self-couplings (on the diagonal) have a maximal time lag of 0.4 s, cross-couplings have a maximal time lag of 0.1 s. These values were determined by a model selection procedure. **C**, Simulated spike trains from the estimated model reproduce the pyloric rhythm. Spike trains were taken from the experimental recording for one second. Then, spike trains were simulated as a random sample from the point process model. The simulated three-neuron network reproduces the stereotypical pyloric rhythm. **D**, Simulation with PY-to-PD connection forced to zero. If the PY-to-PD connection is removed from the model, the remaining model network still exhibits a pyloric rhythm. Spike trains obtained from experiments were used for one second, afterward spikes were simulated using the maximum-likelihood fit of the model. **E**, The strengths of all six directed couplings are plotted as a function of the length of the data set used for fitting the model. **F**, Coupling strengths as a function of the minimal parameter value. Relative strengths remain invariant for all reasonable choices of 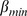. The value used throughout the analysis is indicated by a vertical, dashed line. **G**, Results obtained from the point process model are reproducible across independent data sets. Data set 1 corresponds to the data set used in all analysis and other subpanels, except where otherwise noted. Inferred network strengths are shown for three additional preparations. For all data sets, the physiologically nonexistent connection is weakest. Horizontal scatter is for visualization only.

In point process models, the spiking activity of a neuron is conditionally explained by the previous firing activity of the neuron and activity of the recorded population (see Figure 2A for an illustration). Each neuron's previous spiking contributes to its predicted activity through self-coupling filters and the firing of other neurons in the population contribute with (possibly distinct) cross-coupling filters. All contributions are linearly summed and transformed into an instantaneous firing probability via a sigmoidal, nonlinear transfer function. We define coupling strength here as the net area under the (directed) cross-coupling filter. This implies that a strong coupling could be obtained either by a consistent influence of one neuron to the target neuron over an extended period of time or via a strong, but timely interaction.

We fit such point process models to an extended recording of spontaneous activity of the pyloric circuit and obtained highly significant values for all possible cross-interactions. Hence, judging network structure only from the statistical significance of the model parameters did not reveal relative coupling strengths (see below for a more detailed analysis using the Granger causality approach). The coupling filters used by our model can be interpreted as synaptic-like interaction filters (Figure 2B). Here, negative (positive) values indicate an effective inhibitory (excitatory) effect on the spiking probability at the specified delay. Self-coupling filters (Figure 2B, panels along the diagonal) show three features: an initial refractory period, a rapid transition to a positive peak due to the natural bursting activity of the spike trains, followed by an extended effective inhibition.

The magnitude and time scales of these features can be mapped to biological findings. For example, the positive peaks at lags between 20 ms (LP) and 50–60 ms (PD and PY) directly correspond to the typical inter-spike intervals within the bursts. The spike-triggered depolarization of the membrane potential is on the same time scale and can be measured with intracellular recordings [35]. The following spike rate adaptation is on the order of 200 to 400 ms and longer (Figure 2B, panels along the diagonal), consistent with reported (short) adaptation time scales of 200–300 ms [35], [36]. Hartline also reported adaptation on longer timescales (3–4 s) which is consistent with the shape of the self-coupling filters in models for which longer time lags are considered.

The six cross-history kernels (off-diagonal panels) can be separated into two groups: couplings in the direction of the firing order during the pyloric rhythm (PY-to-PD, PD-to-LP, and LP-to-PY) and couplings counter to the order of a pyloric cycle (LP-to-PD, PY-to-LP, and PD-to-PY) (Figure 2B). The first group has weak to moderate inhibitory coupling, the second group is inferred as strongly inhibitory over the whole range of examined time lags because no spikes are observed in the target neurons during the time lags.

The net interaction type of all inferred cross-couplings is inhibitory, in accordance with known synaptic properties of these neurons [31]. Notably, the only connection not present in the biological circuit (PY-to-PD), is the weakest one inferred by the point process model. Therefore, by applying a threshold based on *a priori* knowledge of the approximate expected density of the network (i.e., based on the expected number or strength of synaptic interactions), a connectivity diagram can be obtained, matching the known circuit connectivity (Figure 1B, lower right).

Not only is the physiologically absent connection the weakest in the model estimate, the relative strengths of the other couplings qualitatively match the known physiology: Experimental studies of directly measured IPSPs between all coupled pairs have revealed a qualitative distinction of synaptic strengths between “weak” and “strong” synapses. For the specific three-neuron circuit (PD, LP, and PY) considered here, the LP-to-PY coupling is considered weak, while all other connections are considered strong [35]–[38]. This is in agreement with our results (Figure 1B, lower right, and Figure 2B). For the time scales of some interactions (LP-to-PD, PY-to-LP, and PD-to-PY) we can only extract lower bounds based on the model fit, but the order of magnitude matches with what is known from physiology for these specific connections (time scales of 80 ms [35] and longer [37]). The shape of the inferred couplings from the PD to the LP unit shows a time scale of approximately 50 ms, consistent with reported values (70 ms [35], [38]). The time scale of the LP-to-PY connection is with approximately 20 ms in close agreement with experimental findings (20–40 ms [35], [36]).

To characterize how well our model fits the observed spiking pattern, we used the model to generate simulated spiking activity following a period of observed spikes. We find that stochastic simulations from the model generally produce a spiking pattern qualitatively similar to the pyloric rhythm observed in the real data set (Figure 2C). In spite of the involved stochasticity in simulating novel spiking activity from the model, the rhythm is accurately maintained for arbitrary periods of time (Figure 2C, the pyloric rhythm was maintained for at least 500 s in 9 out of 10 stochastic simulations). The mean burst Fano factor of the stochastic sample is 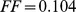 and much smaller than 1, consistent with the statistics of the real spike trains.

Although the model assigns a nonzero value to the PY-to-PD coupling, it is not essential to produce or maintain the pyloric rhythm expressed by the model circuit. To demonstrate this, we set that particular cross-coupling filter to zero in the maximum-likelihood fit and left all other parameters unchanged. When we used this modified model to simulate new spike trains, it displayed a triphasic rhythm (Figure 2D) qualitatively similar to the one obtained using the full model (Figure 2C) or even the recorded activity (Figure 1A, left). Thus we conclude that the estimated PY-to-PD coupling is negligibly weak so that we can correctly predict it to be missing from the biological circuit.

### Inferred connectivity with a point process model is robust to parameter changes and can be replicated across independent data sets

First, we show robustness to the amount of data used for fitting. Specifically, we fit a sequence of models with increasing amounts of data used to train the model and observed the evolution of coupling strengths over time (Figure 2E). We found that the particular connection (PY-to-PD), which is absent biologically, consistently possesses the weakest coupling strength among all six inferred edges. In general, all estimates of coupling strengths remain relatively robust with regard to the length of data analyzed. Specifically, the difference between the mean coupling strength calculated using half of the data compared to using the full data is not significantly different from zero (paired t-test, 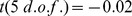, 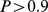). Convergent coupling strengths can be obtained from 30 s or more of spiking data.

Model parameters 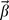 were fitted using standard maximum-likelihood techniques. Prior to fitting, explanatory variables that perfectly predicted the absence of spikes were removed together with the corresponding data bins. Their maximum-likelihood coefficients diverge to minus infinity, so we set them to 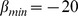. This ensured the resulting probability of spiking to be practically zero. Relative coupling strengths remain unchanged for all sensible values of the cut-off parameter (Figure 2F). Therefore, our results are robust to changes in the value of 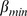.

It is known that the maximal time period to consider history effects can have a profound effect on the inferred networks, for both linear and point process models. For the point process model considered here, the maximal time lags for the self- and cross-coupling filters were not chosen arbitrarily, but based on a model selection procedure that selected an optimal time scale based on a penalized likelihood criterion (Figures S1A and B).

To investigate whether the difference between the weakest (PY-to-PD) and the remaining connections was significant, we computed the uncertainties associated with the coupling strengths based on the maximum-likelihood estimate of the model and its covariance structure (see Text S1). The standard deviations show that the PY-to-PD connection is significantly weaker than any other connection (effect size 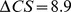 in standardized units; one-sided z-test for the difference between the weakest and second-weakest connection, 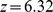, 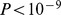 ; Figure S1C).

We also performed a goodness-of-fit test tailored to the point process model based on the multivariate time-rescaling theorem [39]. While the individual fit to the PD neuron is formally rejected at a significance level of 5%, overall goodness-of-fit indicates a reasonable model fit. Furthermore, goodness-of-fit tests performed on the joint spike train of all three units do not suggest a major model misspecification (see Figure S2 and Text S1 for details). Passing all multivariate tests increases our confidence that the dependency structure of the network is being correctly inferred.

Finally, we repeated the model selection and fitting procedure for three additional independent preparations from different animals, each with spike train recordings of variable length. All recordings qualitatively showed a stable pyloric rhythm, although the temporal scales, like the burst cycle period and the exact temporal phase relationships between units, varied considerably across data sets.

For all four data sets, we found qualitatively similar results regarding the inferred connection strengths (Figure 2G). Notably, for all network patterns, the biologically nonexistent connection is inferred to be the weakest compared with all possible connections. Furthermore, relative connection strengths are comparable across all four data sets and filter shapes showed similar qualitative features (not shown). This finding indicates an additional robustness of the presented analysis approach, namely that the same network pattern can be observed in independent preparations.

### An alternative definition of coupling strength based on Granger causality fails to reconstruct the known physiological connectivity

Kim et al. and others used a measure based on Granger causality to quantify the effective coupling between spike trains [22], [23]. The Granger causality score quantifies changes in model likelihoods that reflect statistical significance of couplings rather than a functional interpretation. The Granger causality (GC) score for a directed connection between neuron X and Y is derived by comparing the relative predictions of two nested models: If we improve the accuracy of prediction of a model that only uses Y's and other neurons' histories by additionally incorporating the activity of neuron X, the GC score will be significantly different from zero. Granger causality scores are always non-negative and do not distinguish between excitatory and inhibitory couplings.

**Figure 3:**
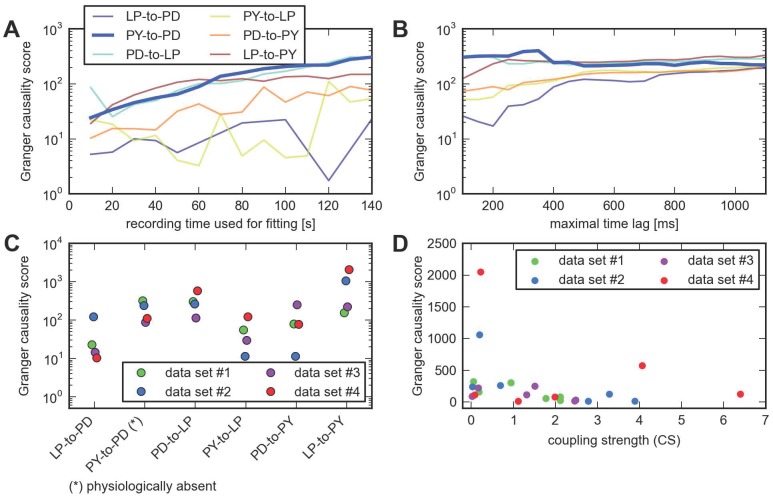
Using a Granger causality score with the point process model does not recover the physiological connectivity. **A**, Granger causality (GC) scores as a function of time used for fitting. **B**, GC scores as a function of the maximal time lag used for fitting (same color scheme as in **A**). **C**, Network inference for all four data sets, using the Granger causality score. The physiologically nonexistent connection does not correspond to the weakest one in any case. Horizontal scatter is for visualization only. **D**, Coupling strengths (CS) and Granger causality scores (GC) are uncorrelated for the point process model. For the point process model, the strength of the coupling can be either defined by the net integral of the interaction filter (horizontal axis) or by the statistical Granger causality score (vertical axis). The scatter plot shows the six cross-couplings for each of the four data sets. The two measures of coupling strength are not significantly correlated (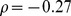, 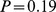).

We used this Granger causality measure using the same point process model as above and parameters determined by the model selection procedure and failed to obtain couplings consistent with known physiology (Figure 1B, lower left). Neither by varying the length of data used for fitting (Figure 3A) nor by varying the maximal time lag of cross-coupling filters (Figure 3B) were we able to yield a network pattern compatible with the known physiology. This conclusion holds true for all four data sets (Figure 3C). In general, we find no significant correlation between the Granger causality scores and coupling strength (CS) defined as the net area under the interaction filters (Figure 3D).

### A linear rate model is insufficient to reconstruct the circuit diagram

**Figure 4:**
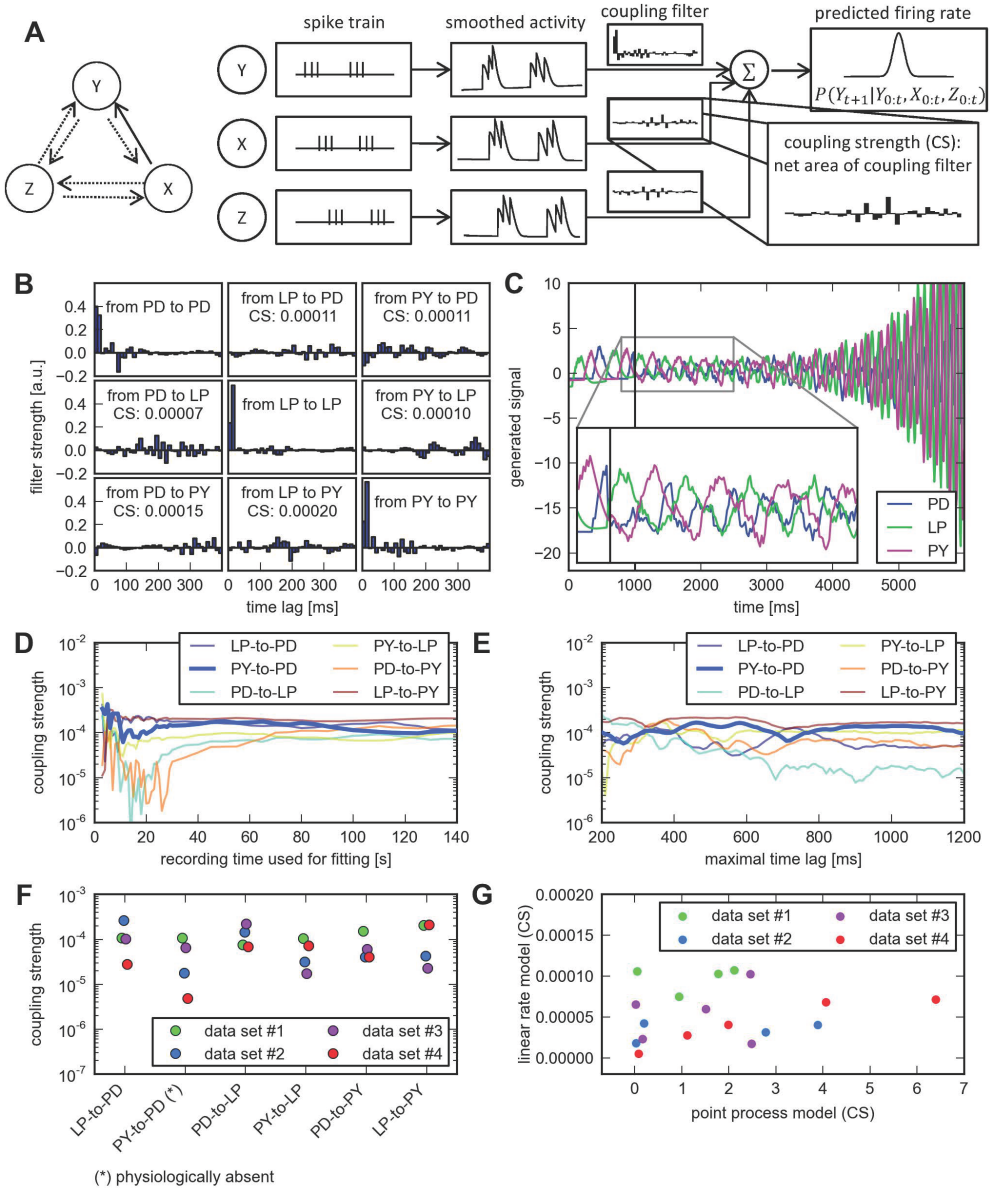
A linear firing rate model is insufficient to reconstruct the correct connectivity. **A**, Schematic overview of the linear rate model. First, spike trains are convolved with a smoothing filter to obtain smoothed time series of firing rates for all neurons (here denoted by X, Y and Z). Linear models are estimated for each neuron (Y) by including auto- and cross-regressive terms from the filtered input of putatively presynaptic neurons (here, X and Z). The firing rate is assumed to be Gaussian with the linearly predicted mean and fixed standard deviation. **B**, Estimated interactions. Coupling coefficients for the auto- (diagonal) and cross-regressive terms (off-diagonal) of the linear model are shown. The maximal time lag was chosen to be 400 ms to match the model of Kispersky et al. [29]. Coupling strength is defined as the net area under the interaction kernel. **C**, The linear rate model fails to generate pyloric-like activity. Neural activity was simulated from the fitted model. First, model output was clamped to the observed activity traces for one second (vertical line). Subsequent activity was simulated using the predictions of the model and a stochastic realization of the noise term. The triphasic burst rhythm is not maintained and modeled neural firing rates diverge after a few seconds of simulated time. **D–E**, The linear model does not accurately reproduce the known physiological connectivity for a wide range of parameter choices, such as the length of the data set (**D**) or a maximal time lag different from 400 ms (**E**). **F**, Network inference using the linear model for all four data sets. The physiologically nonexistent connection corresponds to the weakest one in only two out of the four cases. Horizontal scatter is for visualization only. **G**, Coupling strengths (CS) for the point process model (horizontal axis) and linear rate model (vertical axis) are uncorrelated. The scatter plot shows the six cross-couplings for each of the four data sets. The coupling strength for the two models are not significantly correlated (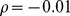, 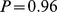).

One might wonder whether a linear rate model as (implicitly) used in [29] combined with our definition of coupling strength might recover the known network architecture. To this end, we constructed a multivariate linear firing rate model as in Kispersky et al. [29] (see *Materials and Methods* and Figure 4A for an overview).

The analysis yielded nine couplings (self-couplings included) between the three neurons (Figure 1B, upper right). All self- and between-neuron couplings had highly statistically significant coupling strengths (Figure 4B). Visual inspection of the coupling filters offered little insight as to whether a potential coupling could be classified as inhibitory or excitatory, and what relevant time scales of the interaction would be.

To test whether the linear model provided a good fit to the data, we used the estimated model to simulate activity after a period of observed activity. If the model were appropriate, we would expect it to produce qualitatively similar spiking activity consistent with the observed data. Instead, we found the linear model is unable to maintain the pyloric rhythm, and activity values start to diverge after only two seconds of simulated activity (Figure 4C). While the linear model qualitatively captures the alternating activation of the three units, it fails to predict any stationary activity. Moreover, the burst-like structure of the spiking activity and the fine temporal relationships between bursts are lost as soon as model output is no longer directly computed from the observed data (Figure 4C, inset). Thus, stochastic sampling from the model produces activity whose statistics are very different from the training data - a general sign of model misspecification. A more detailed goodness-of-fit analysis confirms this suspicion (see Text S1 and Figure S3) and provides evidence that the linear model is insufficient to accurately describe the statistics of the actual recordings.

A further exploration of the parameter space, similar to the previous section, shows that no parameter choice, such as the amount of data used and how far the coupling filters extend in time, leads to a network that would be consistent with physiology (Figures 4D and E). Overall, this indicates that the specified coupling in the linear model is not capturing the true dependency structure of the neurons. In addition, we varied the two remaining free parameters of the linear model: 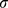, the kernel bandwidth to obtain smooth rate estimates from the spike trains, and f, the sampling frequency of the time series. None of the parameter configurations led to the inference of the physiological network architecture (results not shown).

When we analyzed all four data sets, the physiologically nonexistent connection corresponds to the weakest one in only two out of the four cases. In addition, coupling strengths grouped by connection across all four networks did not show a consistent pattern (Figure 4F, compare to Figure 2G). Moreover, coupling strengths derived from the point process model and the linear rate model were uncorrelated (Figure 4G).

For completeness, we reproduced the original analysis of [29] that used the linear rate model together with the Granger causality measure (Figure 1B, upper left). The failure to retrieve the physiological connectome is independent of the definition of coupling strength (see Figure S4). The analysis demonstrates that although Granger causality estimates can be highly parameter-dependent, the physiological network pattern was not among any network patterns identified for any combination of parameters. Therefore, the failure to recover the correct connectivity in this framework was not due to an inappropriate choice of parameters. Instead, it was caused by intrinsic limitations of the analysis for the type of data considered here. In agreement with the conclusions of [29], the linear rate model is not an appropriate tool to accurately infer the known physiological connectivity of the pyloric network.

### The point process model predicts changing effective networks due to pharmacological manipulation

To this point, we have considered the standard pyloric rhythm in its default configuration. A useful method of network inference should also detect and track changes that occur to the coupling strengths. To this end, we applied the point process model to two data sets where the isolated pyloric circuit is perturbed by pharmacological agents.

**Figure 5:**
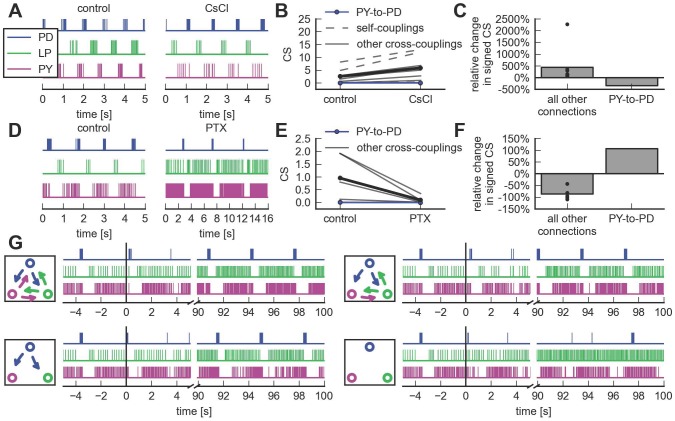
The point process model predicts changes of synaptic coupling strengths due to pharmacological conditions. **A**, Exemplary spike trains from the control condition (left) and after application of CsCl, which blocks the h-current in all neurons (right). The pyloric rhythm is maintained in both conditions. **B**, Inferred coupling strengths for the control (left) and CsCl condition (right). All coupling strengths (self- and cross-couplings) become stronger, that is, more inhibitory. The mean of all eight coupling strengths (thick line, all except PY-to-PD) increases significantly between the control and CsCl condition. The nonexistent PY-to-PD coupling remains the weakest coupling in both conditions (blue line). **C**, Relative change of (signed) coupling strengths between the two conditions. Same data as in **B**, but expressed as the change of coupling strength relative to the control condition. All couplings become more inhibitory with a mean relative change of 450% (left). The relative change of the PY-to-PD coupling has the opposite sign (right). **D**, Exemplary spike trains from the control condition (left) and after application of PTX, blocking glutamatergic synaptic transmission (right). The pyloric rhythm is qualitatively maintained in both conditions. **E**, Inferred coupling strengths for the control (left) and PTX condition (right). All coupling strengths become weaker, that is, less inhibitory. The mean of all five existing cross-couplings (thick line) decreases significantly between the control and PTX condition. The nonexistent PY-to-PD coupling remains the weakest coupling in both conditions (blue line). **F**, Relative change of coupling strengths between the two conditions. Same data as in **E**, but expressed as the change of coupling strength relative to the control condition. All couplings become weaker with a mean relative change of −85% (left). The relative change of the PY-to-PD coupling has the opposite sign (right). **G**, Spike trains generated from the model of the PTX data set with various network constraints. Spike trains obtained from the PTX condition were used for five seconds, afterward spikes were simulated using either the full model (left top), a model with the PY-to-PD link forced to zero (right top), a network structure allowing only for non-glutamatergic synapses (left bottom) or a model with no cross-interactions (right bottom). All models except the last one produce spike trains comparable to the real data. The model without any cross-interactions show burst-like and tonic activity but neurons do not fire with a fixed relative phase.

In the first data set, CsCl was applied to a preparation of the pyloric circuit of the STG. CsCl is known to block an intrinsic current, the h-current (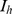), in all cells [40]. The 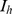 current is an inward depolarizing current that slowly activates upon hyperpolarization of the membrane potential [41]. The spike train statistics show that blocking the h-current has little qualitative effect on the pyloric rhythm generated by the circuit (Figure 5A). This is in agreement with previous experimental reports [40], although we observe changes in individual bursting properties: The burst cycle period increased and overall firing rates of the three neurons were reduced, that is, each burst contained on average less spikes than in the control condition. Firing rates were otherwise stationary within the control and CsCl condition.

We expect that blocking 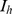 would affect the coupling filters in our model in two ways: First, in both conditions, the PY-to-PD coupling is physically nonexistent and therefore, its inferred coupling strength should be the weakest among all estimated couplings. Second, all other coupling strengths should increase. This is because 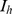 is an inward current that counteracts inhibitory (hyperpolarizing) synaptic coupling from other neurons. Blockade eliminates the post-inhibitory rebound and reduces the likelihood of spikes being triggered after inhibition. Hence, blocking the cell's intrinsic h-current should effectively amplify incoming inhibitory couplings. The same reasoning would predict a strengthening of all inhibitory components of the self-coupling filters, such as the fast component responsible for the refractory period.

Fitting the point process model to the control data set shows a similar pattern as the other four preparations considered so far (Figure 5B). Particularly, the PY-to-PD connection strength is estimated close to zero and is overall the weakest link in the inferred network. After application of CsCl, all physically present coupling strengths increased their magnitude significantly (mean change in coupling strength for all couplings except the PY-to-PD coupling strength: 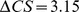, Wilcoxon signed rank test, 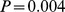 ; Figure 5B and relative changes in Figure 5C). This difference should be contrasted to the change in the (nonexistent) PY-to-PD coupling whose change between the two conditions is two orders of magnitude smaller and in the opposite direction (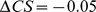). Therefore, although blocking the intrinsic h-current has no immediate effect on physical synaptic transmission in the network, the predicted modulation of coupling strengths is consistent with the observed changes.

For the second data set, we considered a pyloric circuit before and after application of picrotoxin (PTX). PTX is known to block inhibitory synaptic transmission in the STG [42] and affects the functional pyloric rhythm (Figure 5D). When PTX is applied, LP and PY units fire nearly tonically and for longer time periods during a pyloric cycle and partly overlap with firing activity of the PD unit. Overall, firing rates were otherwise stationary in the two recordings.

In the STG, most synapses of the LP and PY cells are inhibitory and mediated by glutamate [43]. Synapses of the PD cell use cholinergic neurotransmission. However, the PD neurons are electrically coupled to AB cells which in turn project to the LP and PY neurons via glutamate [31]. Assuming the AB neuron's activity matches the observed PD activity and is left intact in the preparations, the coupling filter originating from the PD neuron summarizes the joint synaptic effects from the PD/AB group [30], [43]. Therefore, all of the five physical cross-couplings are (partly) due to glutamatergic neurotransmission and we hypothesize the application of PTX should decrease the coupling strengths for all of these connections. The inferred coupling strength of the nonexistent PY-to-PD link should remain close to zero and unaffected by application of PTX.

Indeed, when we fit the point process model to the data sets before and after application of PTX, we find cross-couplings are decreased toward zero, i.e., become weaker (Figure 5E). Notably, the PY-to-PD link remains the weakest coupling strength in both conditions, as predicted. The decrease in strength of the five physical synaptic interactions is significant (Wilcoxon signed rank test, 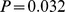) and its absolute effect size (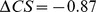) is two orders of magnitude bigger than for the only nonexistent link (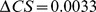, Figure 5F).

To find out which couplings in the network are crucial for the presence of the stable pyloric rhythm, we simulated spike trains from four different models estimated from the PTX condition. The models differed in the constraints placed on the allowed network pattern. As before (Figures 2C and D) experimental spike trains of the PTX condition were used for five seconds, afterward spikes were stochastically simulated using either the fully connected model network, a model with the PY-to-PD link forced to zero, a network structure allowing only for non-glutamatergic synapses or a model with no cross-interactions at all (Figure 5G, from left to right and from top to bottom). All models except the uncoupled one produce spike trains comparable to the real data. The model without any cross-interactions shows burst-like and tonic activity but neurons do not fire in a stable relative phase. This demonstrates that the point process model captures the physiological changes induced by PTX, i.e., the effective network connectivity is reduced to the (weaker) PD-to-LP and PD-to-PY links with all other couplings being effectively absent. In a network with only one synaptic connection or in a fully disconnected network, neurons with temporal irregular activity cannot maintain their relative phase relationships (Figure 5G, bottom right). Therefore, the network with two synapses is the minimal circuitry to maintain the pyloric rhythm (Figure 5G, bottom left), consistent with the experimental findings [37], [38].

Overall, these results illustrate the utility of the point process model in inference of effective connectivity. Bath application of two pharmacological agents alter the expected circuit connectivity by changing either the intrinsic currents of each neuron (CsCl) or the synaptic interactions between neurons (PTX). In both cases, the point process method detected the anticipated changes.

## Discussion

In this work we considered the application of a point process model to infer connections of a three-neuron circuit. To the best of our knowledge, these results provide the first successful application of a network inference algorithm to spike train data recorded from identified neurons within a circuit whose underlying synaptic architecture has been fully characterized. Typically, such inference algorithms have only been validated using simulation studies [15], [20], [23], [26], [27], [44], [45]. We have also shown how measures of effective connectivity can be useful in characterizing the effects of pharmacological treatments on the network connectivity.

### The crab stomatogastric nervous system as a model system for network inference

The crab stomatogastric nervous system is well suited to study network inference algorithms like the point process model. The circuit consists of a small number of elements whose synaptic interactions are well studied and whose monosynaptic connectivity is established [30]. Furthermore, one can routinely and concurrently record from the important units of the circuit. Despite the small size of this network, the rhythmic activity of the neural elements makes it challenging to infer the correct, causal relationships [29].

Most of our analysis can be readily applied to other small neural circuits, e.g. central pattern generators in the respiratory system in vertebrates [46], [47] or motor systems in invertebrates [48], [49], as well as to recordings of larger populations. When applying effective network analyses like point process models to any circuit, the challenge of assigning action potentials to single neurons (spike sorting) arises. Identifying single spiking events and the accuracy of categorizing them as arising from distinct neurons becomes increasingly challenging in recordings of larger neuron populations. Fortunately, in the STG individual pyloric neurons can be recorded on separate nerves making spike sorting trivial. We note that, in general, efficient spike identification is a requirement for the success of any network inference method like the one presented here.

Synaptic transmission in the STG occurs as a graded (analog) release of neurotransmitters and is thus mediated by sub-threshold depolarizations as well as spikes [50], [51]. Therefore, spikes are not the major source of transmitter release, but are dominantly used to signal to the muscles over long ranges. It is not evident *a priori* that a model that treats the time of spikes as the sole input, i.e., does not have access to the membrane potential, can correctly perform connectivity inference. For the circuits considered here, this did not seem to pose a problem because, at least in the STG, prolonged membrane depolarizations always appear simultaneously with spiking activity. Therefore, spikes are proxy measurements to determine the state of the membrane potential. Furthermore, the time scales of the graded synaptic interactions are similar to the ones observed from spike-triggered transmitter release and the ones estimated in our model [51], [52]. In other circuits where graded transmission does not correlate with spike times, knowledge of the subthreshold voltage activity of the neurons might be necessary to infer structural circuit information. We note that synaptic transmission in cortical networks is heavily dependent on spike-triggered, chemical transmission, so the proposed method does in principle generalize to these data.

A model of the central pattern generator for the pyloric rhythm can be evaluated using at least two criteria: One criterion is how close the model reproduces a given, observed set of spike trains and their statistics, e.g., the number of spikes per burst and the average inter-burst duration. For understanding the functional behavior of the circuit, a broader criterion can be applied: A model would match the data if it qualitatively reproduces the stable, triphasic burst pattern, regardless of the exact spike train statistics. It is evident that many models will fulfill either one or both criteria with the first criterion being an additional constraint on the second. This explains why deviations from the best-fitting model (according to the first criterion) can still generate spike patterns that may be equally functionally valid (e.g. by enforcing a certain network structure different from the physiological or fully connected case, see Figure 5G).

Finally, although the pyloric network generates a triphasic motor pattern, these cells are part of a larger circuit, the stomatogastric ganglion of the crab; and the inferred connections are potentially confounded with indirect (cascade) synaptic effects or unobserved common input [53]–[55]. In general, there is unlikely to be a confound in the specific case of the pyloric circuit because the three observed units (PD, LP, and PY) are sufficient for generating and maintaining a pyloric burst rhythm *in vitro* [31]. In principle, an effective coupling from the PY to the PD unit could be realized by a polysynaptic pathway through the inferior cardiac (IC) neuron [31]. This would render the potentially observed coupling as effectively excitatory. However, we found no evidence for an effectively excitatory PY-to-PD coupling in our analysis, indicating a small magnitude of such second-order effects for this circuit analysis. Furthermore, analysis of recordings that included the activity of the IC neuron showed that inferred couplings were not significantly altered by the rhythmically active IC neuron (results not shown).

### Limitations of previous approaches

To elucidate the reasons why Granger causality analysis using a linear model failed to recover the true connectivity in [29], we applied a series of goodness-of-fit tests to identify model misspecifications. We identified that two major changes are necessary for correct inference: First, the use of a nonlinear point process model instead of a linear rate-based model, and second, an alternative definition of coupling strength based on the net area of the coupling filter instead of a reliance on statistical significance. We will now discuss these two aspects in detail.

Analysis with an underlying linear rate model is based on the assumption that neural firing rates are linearly interacting. Even the inclusion of very long time scales in the linear model did not lead to a correct inference using any of the proposed connectivity measures. This observation points to a general limitation of the simple linear autoregressive models. Further, the physical mechanism for the LP and PY neurons to initiate spiking is a release from inhibition [56]. This mechanism cannot be sufficiently captured by a linear model because a strong inhibition would predict negative firing rates and thereby increase the mean-squared error of the predicted activity - the criterion that linear models try to minimize. The biophysical mechanisms that govern the rhythm are highly non-linear, too. By contrast, the nonlinearity in the point process model has more flexibility in modeling inhibitory interactions (including modulated release from inhibition, e.g., via application of CsCl). Both the linear and nonlinear models are multivariate, i.e., they condition the directed couplings based on all other observed network activity.

A firing rate model includes a smoothing preprocessing step on the input spike trains. When applied to data from the STG this preprocessing preserves the qualitative phase relationship between the neurons during the pyloric rhythm, but temporal information about the spike timings is lost. In a system that relies heavily on graded synaptic transmission, like the STG [50], [51], this may not result in a loss of information. However, in networks where spikes causally affect the postsynaptic membrane potential, we expect that fine temporal relationships between spikes and postsynaptic activity (or absence thereof) are predictive of synaptic coupling. Here, we circumvented the smoothing step by proposing a point process model that explicitly models neural data as a sequence of events in time.

We have observed that the amount of available data strongly influenced the statistical significance of a directed coupling. Typically, for our data sets, 15 seconds of data or more are enough to yield highly significant Granger causality (GC) scores for all six connections, for both the linear and point process models. Hence, statistical significance alone is not useful in this case to determine the presence of effective coupling between two neurons. Because the magnitude of the GC score varies as well with the amount of data, its value cannot be used for inference of coupling strength beyond relative comparisons (see also [57]). Moreover, Granger causality scores were practically uncorrelated with our proposed measure of coupling strength. This is because Granger-based significance analysis strongly depends on the amount of data used and absolute values of inferred GC coupling strengths are difficult to interpret. In the statistical framework proposed here, inference is based on the effect size of an inferred coupling rather than statistical significance. We propose defining coupling strengths as a property of the estimated filters. This allows better interpretability of the results and the separation of coupling strengths between the nonexistent biological connections and the remaining ones.

Granger causality analysis and related approaches are sometimes called *model-free* procedures [45], [58]–[60], but are still based on implicit model assumptions. These assumptions are rarely checked in practice, and the final GC scores and their P-values are commonly the only factors used for inference. For the proposed point process model and functional definition of coupling strength, by making the model assumptions explicit, we allow for the application of rigorous goodness-of-fit and model selection procedures that help in choosing a suitable model.

### The point process model as an example of a statistical inference procedure

The point process model was primarily used as an inference tool to deduce the connectivity between a set of observed neurons. Although this constitutes a statistical and phenomenological model (i.e., it does not explicitly model biophysical processes), we have shown its potential as a generative model (Figures 2C and D). The coupling filters of the point process model have both statistical and physiological interpretations, analogous to biophysically-based synaptic interactions.

Coupling filters interact in a multiplicative way, i.e., they modulate an underlying baseline firing rate instead of increasing or decreasing the firing rate by a fixed amount. The coupling strength (the integrated area under the interaction kernel) is related to the number of spikes that, depending on the instantaneous postsynaptic firing rate, are generated or suppressed on average due to a single presynaptic spike [35]. Because of the sigmoidal nonlinearity between the linear summation of couplings and the resulting firing rate, the effect of a presynaptic spike can vary dynamically depending on the current gain (slope) of the transfer function. Therefore, the model can partly distinguish synaptic interactions from postsynaptic excitability unlike previous approaches.

From a biological perspective, the sum of contributions of past neural activity in a point process model can be interpreted as the influence on the neurons' membrane potential. The coupling filters correspond to synaptic interactions, e.g., in the spike-response model [61]. The shapes of the filters suggest the time scales of the synaptic (or effective) interaction, their sign (excitatory versus inhibitory), and amplitudes. The model is flexible enough to allow for polyphasic responses although our definition of the coupling strength reduces the response to a scalar value (see [37] for examples of polyphasic interactions in the STG). Periodic structure in the spike trains (such as bursting and the time scale of the periodic pyloric rhythm) can be read off from the peaks in the filters at the corresponding time lag because they represent the modulation of the firing probability locked to the exact spike timings. Although the coupling filters have a similar interpretation in the linear model, in the STG analysis, their shapes were not suggestive of the type of interaction.

The relationship between the effective coupling filters and biological postsynaptic potentials is not unique. This is especially true for inhibitory connections in the STG: Consider presynaptic spiking activity that always occurs at a fixed relative phase of the postsynaptic burst cycle in which the postsynaptic neuron is already hyperpolarized. The observation of the absence of any postsynaptic spike does not contain any information about the amplitude of the synaptic conductance beyond a minimal value that prevents the postsynaptic neuron from firing. In these cases, estimates of coupling filters diverge and we cap them at an arbitrary value that does not affect the qualitative results of the analysis. This so-called phase response saturation has been shown in experiments and detailed neuron models of the pyloric rhythm [62], [63] and should serve as a reminder that neural couplings might not be uniquely identified when no information about the subthreshold activity is available.

We note that while using the net integral of the coupling filters as a measure of coupling strength has led to a good correlation between inferred coupling strengths and the presence of real couplings, other measures of coupling strengths might be useful to consider as well. These could include other features of the kernel (such as its peak amplitude) or be limited to certain temporal scales (e.g., near-simultaneous, or short versus prolonged interactions).

For the point process model presented here, all available data were used and free parameters were chosen with a straightforward, but rigorous model selection procedure. Because a nonzero coupling strength is recovered for each possible connection, different binary connectomes can be obtained by varying a threshold that determines whether a connection is substantial. Using a threshold to determine a binary circuit diagram based on *statistical* significance alone would result in the inference of a fully connected network. Yet, we have observed that setting the known missing physiological connection to zero did not change the functional behavior of the modeled circuit suggesting that statistical significance is not an appropriate metric for determining functional interactions in this data set. It is known that networks with different neuron parameters can express very similar pyloric-like rhythmic activity [64], [65]. A more sophisticated procedure that chooses an optimal threshold in a data-driven way based on *physiological* significance is desirable.

Finally, an advantage of point process models is the availability of goodness-of-fit tests that are not always assessed in practice in Granger causality analysis. When we applied model adequacy tests to the linear rate model, we could identify its shortcomings in capturing the structure of the data. The results hinted at the necessary modifications to construct a model whose network inference could match the physiology. Because any model-based assessment of connectivity is expected to show model misspecifications given enough data, we suggest methods that explicitly consider the structure of the data in building the model and use interpretable measures of connectivity rather than statistical significance levels. A series of goodness-of-fit tests, tailored to the point process nature of the model, strengthened our confidence in the model's inferred network structure and demonstrated the robustness of our results.

### Relevance to identification of large-scale networks

In general, effective connectivity will not necessarily be equal to physiological or structural connectivity [66], [67], even if our study suggests sophisticated statistical models might permit inference of actual physiological connectivity from extracellular recordings. Especially for larger-sized (and cortical) networks, effective connectivity between a subset of neurons will be different from physiological connectivity. This is because of indirect connections and shared, unobserved inputs. Nevertheless, because monosynaptic direct couplings should form a subset of inferred effective connections [68], such a measure can still be useful [22], [23], [34], [66], e.g., to improve decoding performance (see [69] for a recent demonstration with multi-electrode recordings in different cortical regions), to distinguish different network states or to track plasticity-induced changes [33], [70]. We have demonstrated, for example, that partial blockade of synaptic transmission strongly reduced the strength of inferred couplings. In addition, changes of intrinsic currents not explicitly represented in our model can be characterized using the notion of an effective coupling between neurons or coupling of a neuron with itself. As such, we expect the class of point process models presented here could also be useful in other contexts of neurophysiology, such as characterizing single-neuron responses [71], [72] or general network dynamics [70], [73].

Although we have shown that linear models do not recover the physiological network architecture in the pyloric circuit, they may be more applicable to large networks where measurements reflect averaged population activity and nonlinearities may potentially average out [12]. Ultimately, to compare the relative performances of the models put forward here, the approach taken in this study must be scaled to larger networks and recordings [74]. Although simultaneous recordings from many neurons are now routine, we lack the necessary independent assessment of their structural connectivity.

Experimental protocols necessary to obtain both signals and structural information of neural circuits are being actively developed: A recent study combined *in vivo* functional imaging using two-photon calcium imaging with subsequent paired patch-clamp recordings of the same individual cells in slices [75]. For a small number of cell pairs, synaptic connectivity could be unambiguously inferred using the intracellular recordings. Progress in multi-photon imaging has been made to achieve the temporal resolution necessary to infer sequences of spikes from such functional recordings [76]–[78]. Taken together, these approaches could be used to validate connectivity inference algorithms based on spike trains or imaging signals in the future [79]–[81].

A growing scientific community is interested in multi-neuron models and connectomics. As these data become more widely available, principled methods that incorporate known statistical structure in the data — such as the one proposed here — will be of fundamental importance.

## Materials and Methods

### Experimental details

Full experimental details for the four data sets can be found elsewhere [29]. Briefly, Jonah crabs (Cancer Borealis) were purchased from a commercial food supplier (Commercial Lobster, Boston MA) and held in artificial seawater tanks at 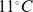. Prior to dissection, animals were put in ice for 30 minutes to numb them. The stomach was removed from the animals and pinned into a dish and immersed in physiological saline containing: NaCl, 440 mM; KCl, 13 mM; MgCl2, 26 mM; CaCl2, 13 mM; Trizma base, 11 mM; maleic acid, 5 mM; pH 7.45. Under a microscope the stomatogastric nervous system (STNS) was separated from surrounding tissues and pinned into a smaller dish for electrophysiological recordings.

Vaseline mixed with mineral oil was used to build waterproof wells around identified nerves to record action potentials from stomatogastric ganglion (STG) neurons. Steel electrodes were placed into these wells with reference electrodes in the bath to record electrical signals. These signals were recorded with an AM Systems Model 1700 AC Amplifier and digitized with an Axon Instruments Digidata 1440A (Axon Instruments, Sunnyvale, CA). pClamp software (Molecular Devices, Sunnyvale, CA), running on a PC computer, was used to record extracellular signals continuously.

During recordings, saline was continuously perfused and recording temperature was kept as close to 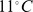 as possible with a Peltier cooling system (Warner Instruments, Hamden, CT; Harvard Apparatus, Holliston, MA). Spikes were extracted for three different neurons (PD, LP, and PY) from three different nerves (pdn, lvn, and pyn), respectively. Single spikes were extracted by a threshold criterion. Spike trains were analyzed off-line using Spike2 software (CED, Cambridge, UK) and then exported to MATLAB for further processing.

Recordings were obtained from four preparations for recording periods ranging between 140 and 300 seconds. Results reported in the text and figures refer to a single data set (#1), unless otherwise noted. Results are qualitatively similar for all four data sets.

For predicting changes of coupling strength by pharmacological conditions, data were acquired in a similar way as described above. Specifically, CsCl at 5 mM concentration was applied to the preparation to block h-currents. Recordings include 300 s of data before the application (control) and 300 s after application of CsCl (condition). Visible spike sorting artifacts were removed by visual inspection. The model selection procedure selected a maximal lag of 1 s for the self-history filters and 350 ms for the cross-coupling filters where maximal lags were jointly optimized for both data sets using the BIC-penalized likelihood criterion.

For the application of picrotoxin (PTX), the control condition consists of 360 s of recordings before the application and 120 s of stationary activity 6 minutes after application of PTX (Sigma Aldrich, St. Louis, MO) at 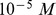 added to the saline. Spike trains were acquired as described previously. The model selection procedure selected a maximal lag of 1300 ms for the self-history filters and 100 ms for the cross-coupling filters where maximal lags were jointly optimized for both data sets using the BIC-penalized likelihood criterion. For the generation of stochastic spike trains from the model, maximal lags for the model of the PTX condition were manually chosen to accommodate the long period of the pyloric rhythm (approximately 5 seconds).

### Point process model

A multivariate point process model of the spiking activity was constructed using the conditional intensity framework [82] for which the instantaneous firing intensity (or rate) 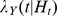 for neuron Y is given by:
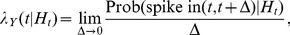
(1)where 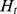 summarizes the activity of all neurons up to time t and possibly other extrinsic variables, and 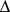 denotes the length of a time period. For a time-discrete model with 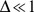, the probability of spiking in a time bin i becomes:
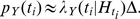
(2)Observed spike trains were converted into a binary sequence of spiking activity 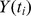 that indicates whether or not there was a spike in the time window 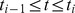. The model can be easily adapted to multi-unit activity (MUA) by replacing the Bernoulli likelihood with a Poisson likelihood that allows an arbitrary number of spikes per time bin. The point process likelihood is approximated by the likelihood of the binary Bernoulli model 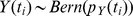 so that the log likelihood of the data is given by:
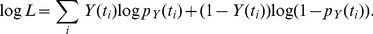
(3)

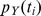
 is modeled as a nonlinear transformation of a linear sum of explanatory variables:
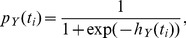
(4)where 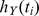 sums the effects of the recent spiking activity of the neuron itself (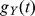), the activity of other neurons (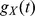 and 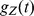) and possibly other factors. Hence, 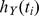 is the sum of these three terms plus a constant baseline:
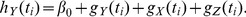
(5)Here, the constant baseline 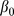 regulates the spontaneous firing activity and 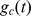 are convolutions of the spike train of neuron c with a coupling filter. Coupling filters are modeled with spline basis functions with knot points separated by 5 ms up to the maximum lag (see [34] for details). Specifically, if 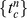 denotes the nth spike time of neuron c and 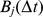 is the jth out of 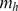 basis functions for a self-coupling filter (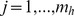), then:
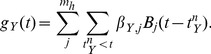
(6)Similarly, the contributions from the cross-coupling terms are given by:
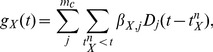
(7)where the basis functions of the cross-coupling filters are denoted by 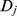, 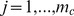 (and similarly for 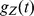). Note that although spline basis functions are used for both self- and cross-coupling filters, 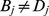 due to different maximal lags (unless 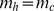). The exact shape of the basis functions, i.e., the order of the spline representation, did not have a significant impact on the reported results (see Figures S1F and G).

We determined the maximal lags for the self- and cross-coupling filters separately by a BIC criterion [83]. For the self-history kernel, models with varying maximal lags (up to two times the burst cycle period of the data set) were fit without any cross-couplings. The burst cycle period is defined as the length of the data set divided by the number of bursts separated by an interspike interval of more than 200 ms. The negative log-likelihood 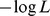 evaluated on the data used for fitting (Equation (3)) was corrected by a term 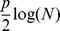 where p is the number of model parameters and N is the number of sample points to yield the BIC value: 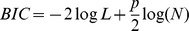. We then summed BIC values for all neurons of the same data set.

Once we determined the maximal lag for the self-coupling filter, we fitted full models including the cross-coupling filters and varied their maximal lag up to 1.2 times the burst cycle period of the data set. The maximal range of tested values was chosen so that a U-shaped curve could be obtained in all cases. The lag that corresponded to the minimal BIC value was then chosen as the maximal lag for all six cross-coupling filters.

Model parameters 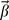 were fitted using standard maximum-likelihood techniques [84], [85]. Prior to fitting, explanatory variables whose presence allowed the perfect prediction of the absence of spikes were removed together with the corresponding data bins. This was the case, for example, whenever spikes of a putative presynaptic neuron were never followed by a spike of the modeled neuron at a fixed delay. The maximum-likelihood solution for the value of the interaction filter at this delay diverges to minus infinity. To ensure convergence of the model estimation procedure, the corresponding coefficients were fixed to −20 so that the resulting probability of spiking is practically zero. Furthermore, a lower bound of −20 was imposed on all coefficients. The results of the analysis are not dependent on the exact value of this cut-off parameter (Figure 2F).

Statistical significance of single parameter values can be (approximately) established using the Wald statistic [85]. Here, we are interested in the statistical significance of a specific interaction filter that is composed of 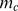 basis functions with associated parameters. If 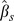 denotes the subset of parameters of the complete estimated parameter vector 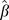 and 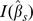 the corresponding entries of the observed Fisher information matrix, then the compound test statistic 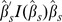 follows (approximately) a 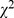 distribution with 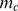 degrees of freedom [85]. In practice, all parameter estimates were highly statistically significant so that the approximative nature of the formula is negligible.

Spike train activity was simulated from the model by drawing stochastic samples according to 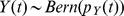 with 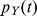 given by Equation (4) and similarly for neurons X and Z. The initial spike-history terms 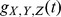 were computed from 1 second of observed spike trains.

We applied the previously described analysis steps to all four data sets. Specifically, the model selection procedure (using BIC-corrected log likelihoods) is performed separately for each data set. We report and visualize the results only for the first data set, unless otherwise noted. For all data sets, we used the complete recording periods unless otherwise noted.

### Linear rate model

Firing rates for the three neurons X, Y and Z are obtained from the spike train recordings by first convolving the spike trains with a half-Gaussian (i.e., causal) filter with standard deviation 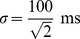. The resulting function is discretized into a time series with sampling frequency of 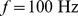. The values of both parameters, 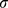 and f, are chosen to be consistent with [29], but we additionally analyzed variations of both parameters in the context of a sensitivity analysis (see Results). Furthermore, linear trends of all time series are locally removed [27].

A multivariate linear model is then constructed for the (normalized, i.e., zero-mean) firing rate at time 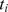 using auto- and cross-regressive terms as follows:
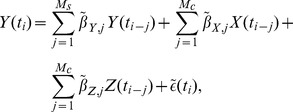
(8)where 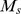 is the maximal time lag to consider for the self-coupling, 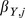 (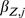) are the model coefficients for the self-interaction (interaction with time-series Z) and 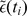 is the noise term. All model parameters were estimated using standard techniques of linear regression. The maximal time lag was set to 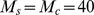 (i.e., maximal lag of 400 ms) [29], unless otherwise noted. A threshold of 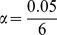 (Bonferroni-corrected for multiple comparisons; six cross-couplings) is used to determine significant interactions.

To generate stochastic samples from the model, multivariate models were first fit to each neuron. For the first second, the time series 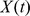, 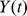 and 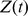 were taken to be the smoothed spike trains of the real recordings and preprocessed as described before. Then, for 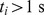, new activity samples were iteratively simulated via Eq. (8) with 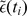 being now an i.i.d. sample from a normal distribution with variance obtained from the model fit.

### Definition of coupling strength

For the point process model, we define the directed coupling strength between two neurons as the net integral of the corresponding cross-coupling filter:
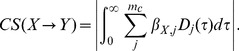
(9)An equivalent definition can be made for the linear rate model. In our case, coupling strengths were qualitatively similar whether a multivariate or only pair-wise model was used (Figures S1D and E).

The type of the directed interaction between X and Y is completely specified by the filter coefficients. The reduction of the (potentially multifaceted) interaction into a single quantity like CS is not unique. For the point process model, Equation (9) captures the integrated modulatory effect of a spike of one neuron onto the spiking activity of the other neuron. We chose the integral of the filter in lieu of, e.g., its peak, because it is a linear function of the model coefficients and thus is more robustly estimated from a finite amount of data. Moreover, potentially polyphasic interactions, such as interactions that are both excitatory and inhibitory on different time scales, are reduced to their dominant mode. An example of such polyphasic dynamics for the self-interaction filter might include short inhibitory refractory effects, followed by excitatory burst-like rebounds and longer suppressive periods.

We use the absolute value of the integral in Equation (9) to obtain a measure of coupling strength that is independent of the actual direction of modulation (excitatory versus inhibitory). This direction of interaction can be assessed by computing 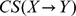 without taking the absolute value: 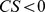 is classified as a net inhibitory interaction, 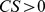 is effectively excitatory. Due to the constraints of the model, CS measures a combination of synaptic interactions and post-synaptic excitability if the latter cannot be completely accounted for by the self-coupling filters, like voltage-dependent ion channel dynamics.

### Granger causality analysis

Granger causality analysis attempts to assess the strength of a causal (i.e., directed) interaction between two time series X and Y in the presence of other explanatory variables, e.g. a third time-series Z. We briefly describe the framework here, more details may be found elsewhere (for linear models, see [27], [86]; for point process models, see, e.g., [22], [23]).

To estimate the causal strength of the directed link 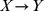, two models are constructed: First, an autoregressive model of Y is built using Y's own history (and the activity of any other explanatory variable, here, the activity of the third neuron Z) to predict its next value. For the point process model, this leads to replacing Equation (5) by:
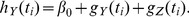
(10)For the linear rate model, the corresponding equation is:
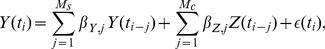
(11)with residual term 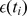, i.e., the difference between the predicted and observed value. In this context, we restrict the analysis to linear autoregressive models with normal innovations, i.e., the residuals are assumed to be independent random samples of a Gaussian distribution.

To assess the interaction 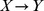, this reduced model is compared to the full, multivariate models as defined above. If the inclusion of X's history significantly decreases the variance of the residuals, there exists a directed link from 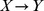 in the sense of Granger causality.

For linear models, the reduction in variance can be measured by the log ratio: 
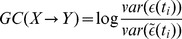
 and its significance can be tested using the F-test procedure (see [27] for details).

For point process models, the Granger causality score is defined by the log-likelihood ratio, or, in other terms, the difference in model deviances [23], [84]. Because the two models are nested and likelihoods are evaluated on the training data, Granger causality scores are always non-negative.

## Supporting Information

Figure S1 **Results obtained from the point process model are robust to changes of parameters.** **A**, Model selection for the maximal lag of self-coupling filters. To determine the time scale for the self-history kernels, models using only self-coupling terms were fitted to the spike trains and the model with an optimal BIC (Bayesian Information Criterion) was chosen (blue dot). **B**, Following the choice of the extent of the self-coupling filter, we added cross-coupling terms to the models of varying lengths and again selected for the optimal time scale according to the model selection criterion (blue dot). The model selection produced self-coupling filters slightly shorter than an average burst cycle period and cross-coupling filters substantially shorter than the burst cycle period so that interactions were deemed important only for time lags on the order of 100 ms. **C**, Variability of parameter estimates. Height of the bars indicate the inferred coupling strength for each of the six possible directed interactions. Error bars denote standard deviation and are obtained analytically from the maximum-likelihood parameter estimates and estimated covariance matrix. **D–E**, Results are independent of the choice of a bivariate or multivariate model. For the linear rate model, using a bivariate (pair-wise) model instead of the full multivariate model increased the absolute value of all Granger causality scores (**D**). In no case is the physiologically absent connection (PY-to-PD, thick blue line) estimated as the weakest. Similar results are obtained when measuring coupling strength as the area under the filter (data not shown). For the point process model, using a pair-wise model versus the full multivariate model has little effect on the inferred coupling strengths (**E**). This is because of the different phases in which the three neurons fire in the pyloric rhythm and the short extent of the coupling filters that lead to little to no overlap between coupling filters. For both versions of the model, the physiologically absent connection is inferred as the weakest (PY-to-PD, thick blue line). **F**, Results do not depend on the choice of the polynomial degree of the basis functions. For the point process model, basis functions for the self- and cross-coupling kernels were chosen as uniform B-splines of order 3, i.e. quadratic degree [87]. Here, the coupling strengths of the six cross-couplings are plotted as a function of different polynomial degrees, ranging from piecewise-constant functions (degree 0), to linear (1), quadratic (2) and cubic degree (3). Relative coupling strengths remain unchanged for all spline orders. Therefore, our results are robust to changes of the basis. The value used throughout the analysis in the main manuscript is indicated with an asterisk. **G**, BIC-corrected log likelihoods are shown for different choices of the polynomial degrees of the kernel representation. Lower values indicate a better fit. The choice of quadratic spline basis functions (asterisk) is justified from a model selection criterion based on the log-likelihood criterion.
(TIF)

Figure S2
**Multivariate goodness-of-fit analysis for the point process model increases confidence of the inference.** **A**, Observed spike train and instantaneous firing rate estimates. Spike trains (top) and estimated firing rates 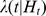 are plotted for the first 800 ms of recordings. Outside of the bursts, the model assigns a zero firing probability to each time bin. Prior to the first spike of each burst predicted firing rates begin to rise when the ongoing inhibition weakens. This should be compared to the linear rate model where modeled activity is nonzero in a broad region around each burst and only slowly decays in the out-of-burst regions (Figure S3A, middle). **B–D**, Multivariate goodness-of-fit analysis. We employed the multivariate time-rescaling test as a goodness-of-fit test for point process models. Residuals are calculated based on the model fit and the observed spike trains and should form a homogeneous Poisson process. Normalized residuals (solid lines) should lie completely within the 95% confidence intervals (dashed lines) to pass the goodness-of-fit test (**B**). Residuals are normalized by the sample size to allow for global confidence intervals. A necessary condition to pass the multivariate time-rescaling test is that the superposition of all spike trains forms a Poisson process with an independent mark sequence. The mark sequence is the sequence of neuron identities which correspond to the spikes in the superimposed process. Independence between consecutive marks in the sequence is tested with a 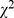 crosstabulation test and shows the residual sequence is compatible with the independence assumption (**C**, 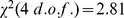, 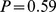). To pass the goodness-of-fit test, the scatter plot of normalized intervals should uniformly fill the unit area (**D**). A 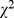 test of independence indicates no significant departure from the independence assumption (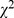 test of serial independence, 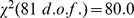, 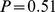, using 10 bins per dimension).(TIF)

Figure S3
**Goodness-of-fit analysis for the linear rate model can reveal its inadequacy.** **A**, Comparison of original and fitted firing rates. The spike trains are smoothed with a half-Gaussian kernel of fixed bandwidth to obtain a smooth estimate of the firing rate (top). The fitted signals of the linear rate models (middle) and the residuals (bottom), the difference between original and fitted signals, is plotted for 150 consecutive time bins. Qualitatively, the observed and fitted activity traces seem to match; however, closer inspection of the residuals reveals that they are neither Gaussian nor white. For a sufficient model fit, residuals should form a sequence of independently distributed Gaussian variables. **B–C**, Residual analysis shows the linear rate model is an inadequate model. The histogram of residuals is shown for the PD neuron (**B**). Lilliefors' procedure rejected the null hypothesis that the residuals are samples of a Gaussian distribution (red line; 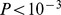). Furthermore, residuals should be uncorrelated over consecutive time bins. A scatter plot of the residuals for two consecutive time bins (**C**) illustrates that residuals are not independent of each other (histogram-based 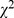 test of serial independence, 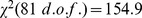, 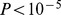, using 10 bins per dimension). Here, residuals are normalized by their empirical cumulative density function. Thus, fundamental assumptions of the linear model, i.e., that the difference between the observed signal and the model is Gaussian and random in time, are violated.(TIF)

Figure S4
**A linear rate model using the Granger causality criterion to define coupling does not accurately reproduce the known physiological connectivity for a wide range of parameter choices.** **A**, Results of linear Granger causality analysis for varying amounts of data used for fitting. For comparison, previous analysis [29] used 5 s. The physiologically absent connection (thick blue line) is never among the weakest connections. **B**, Results of linear Granger causality analysis for varying maximal time lags of the auto- and cross-regressive filters. Previous analysis [29] used 400 ms (vertical dashed line). The physiologically absent connection (thick blue line) is never among the weakest connections. All connections are highly statistically significant regardless of the maximal time lag. **C**, Linear Granger causality fails to recover the known physiological connectivity for all four data sets. Horizontal scatter is for visualization only. The GC scores are not the same as in [29] because we used the full set of recordings. Relative magnitudes are consistent with previous analysis of [29]. Both analyses used a multivariate version of the model. **D**, Granger causality scores for the nonlinear point process model (horizontal axis) and the linear rate model (vertical axis) are correlated (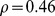, 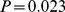). The scatter plot shows the six cross-couplings for each of the four data sets.(TIF)

Text S1 **Additional methods.** We present detailed information about methods on evaluating goodness-of-fit for point process models and linear rate models as well as how to quantify the uncertainty in the coupling strength estimates.
(PDF)
