# Supplementary figures and images for: Successful Reconstruction of a Physiological Circuit with Known Connectivity from Spiking Activity Alone
Source: PLoS Comput Biol. 2013 Jul 11;9(7):e1003138. doi: 10.1371/journal.pcbi.1003138 (PMC3708849; doi:10.1371/journal.pcbi.1003138)

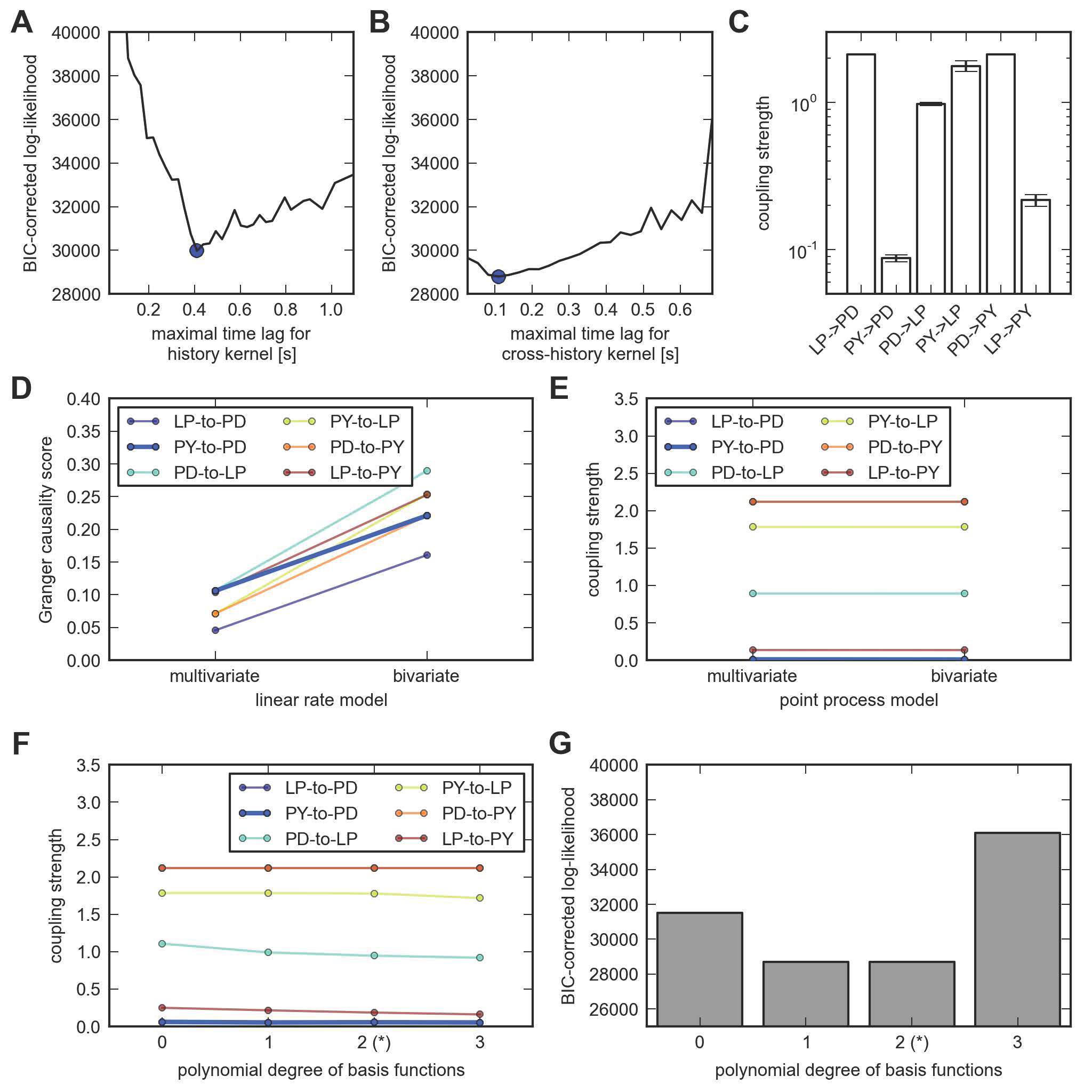

Supplement: Figure S1 — Results obtained from the point process model are robust to changes of parameters. A, Model selection for the maximal lag of self-coupling filters. To determine the time scale for the self-history kernels, models using only self-coupling terms were fitted to the spike trains and the model with an optimal BIC (Bayesian Information Criterion) was chosen (blue dot). B, Following the choice of the extent of the self-coupling filter, we added cross-coupling terms to the models of varying lengths and again selected for the optimal time scale according to the model selection criterion (blue dot). The model selection produced self-coupling filters slightly shorter than an average burst cycle period and cross-coupling filters substantially shorter than the burst cycle period so that interactions were deemed important only for time lags on the order of 100 ms. C, Variability of parameter estimates. Height of the bars indicate the inferred coupling strength for each of the six possible directed interactions. Error bars denote standard deviation and are obtained analytically from the maximum-likelihood parameter estimates and estimated covariance matrix. D–E, Results are independent of the choice of a bivariate or multivariate model. For the linear rate model, using a bivariate (pair-wise) model instead of the full multivariate model increased the absolute value of all Granger causality scores (D). In no case is the physiologically absent connection (PY-to-PD, thick blue line) estimated as the weakest. Similar results are obtained when measuring coupling strength as the area under the filter (data not shown). For the point process model, using a pair-wise model versus the full multivariate model has little effect on the inferred coupling strengths (E). This is because of the different phases in which the three neurons fire in the pyloric rhythm and the short extent of the coupling filters that lead to little to no overlap between coupling filters. For both versions of the model [file pcbi.1003138.s001.tif]

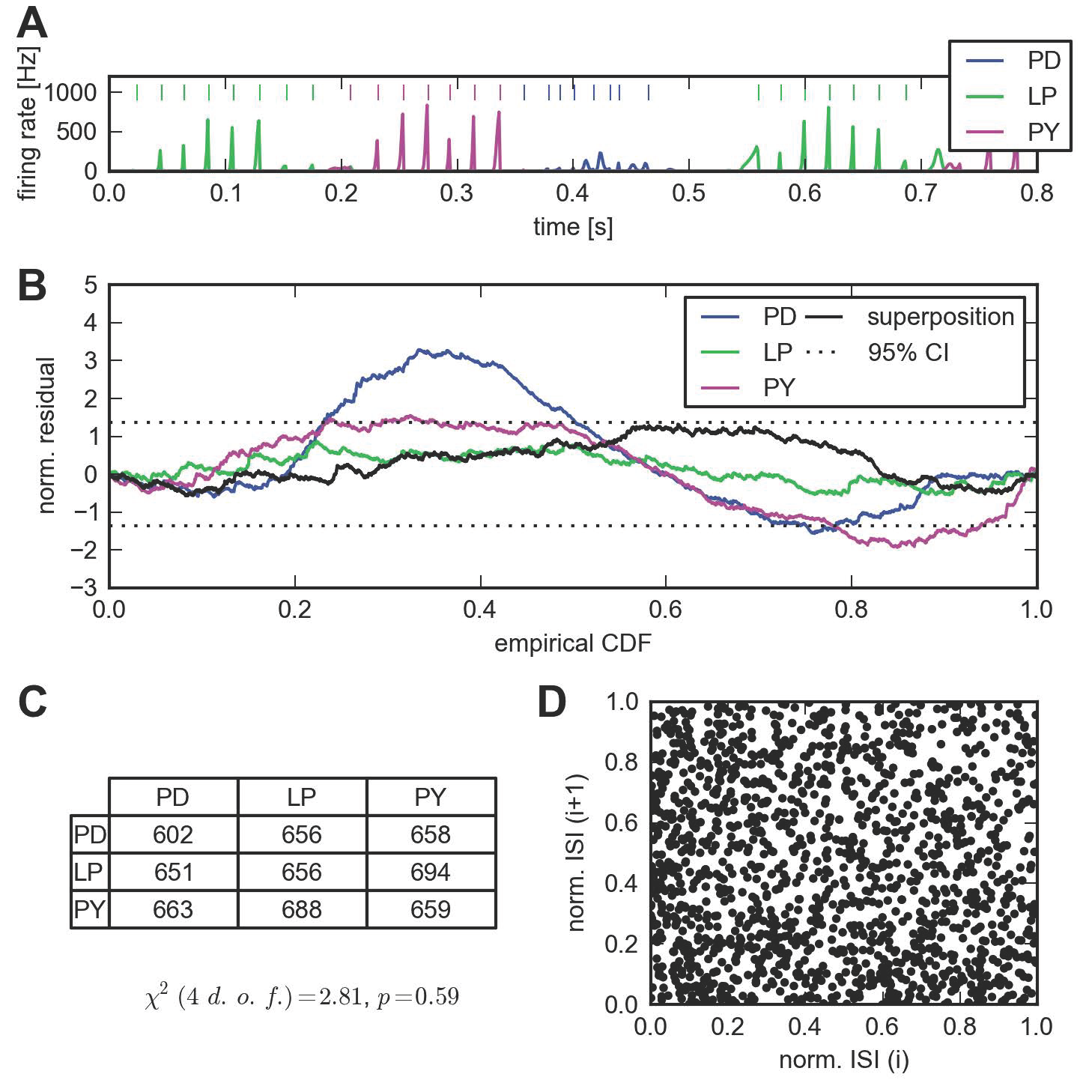

Supplement: Figure S2 — Multivariate goodness-of-fit analysis for the point process model increases confidence of the inference. A, Observed spike train and instantaneous firing rate estimates. Spike trains (top) and estimated firing rates are plotted for the first 800 ms of recordings. Outside of the bursts, the model assigns a zero firing probability to each time bin. Prior to the first spike of each burst predicted firing rates begin to rise when the ongoing inhibition weakens. This should be compared to the linear rate model where modeled activity is nonzero in a broad region around each burst and only slowly decays in the out-of-burst regions (Figure S3A, middle). B–D, Multivariate goodness-of-fit analysis. We employed the multivariate time-rescaling test as a goodness-of-fit test for point process models. Residuals are calculated based on the model fit and the observed spike trains and should form a homogeneous Poisson process. Normalized residuals (solid lines) should lie completely within the 95% confidence intervals (dashed lines) to pass the goodness-of-fit test (B). Residuals are normalized by the sample size to allow for global confidence intervals. A necessary condition to pass the multivariate time-rescaling test is that the superposition of all spike trains forms a Poisson process with an independent mark sequence. The mark sequence is the sequence of neuron identities which correspond to the spikes in the superimposed process. Independence between consecutive marks in the sequence is tested with a crosstabulation test and shows the residual sequence is compatible with the independence assumption (C, , ). To pass the goodness-of-fit test, the scatter plot of normalized intervals should uniformly fill the unit area (D). A test of independence indicates no significant departure from the independence assumption ( test of serial independence, , , using 10 bins per dimension). (TIF) [file pcbi.1003138.s002.tif]

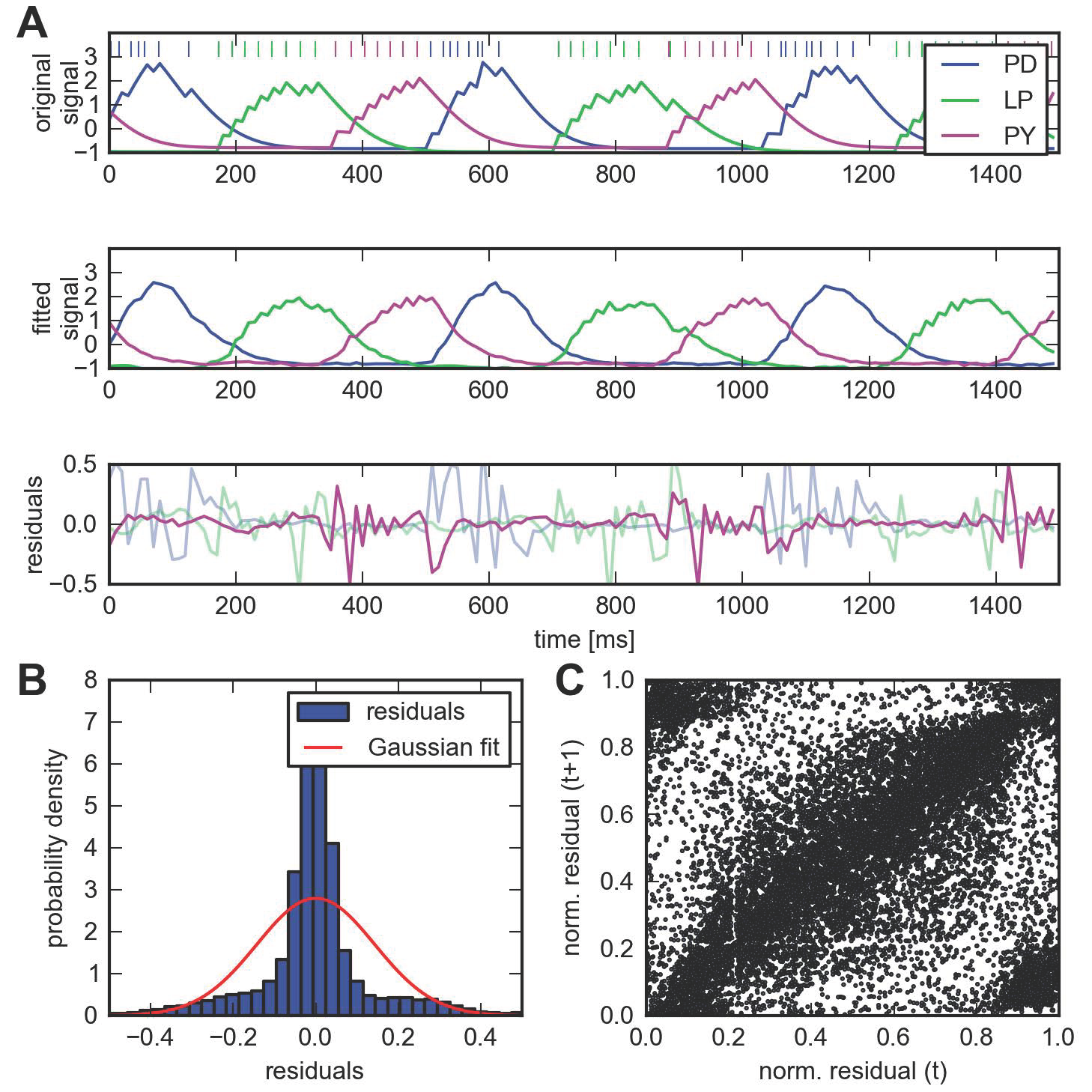

Supplement: Figure S3 — Goodness-of-fit analysis for the linear rate model can reveal its inadequacy. A, Comparison of original and fitted firing rates. The spike trains are smoothed with a half-Gaussian kernel of fixed bandwidth to obtain a smooth estimate of the firing rate (top). The fitted signals of the linear rate models (middle) and the residuals (bottom), the difference between original and fitted signals, is plotted for 150 consecutive time bins. Qualitatively, the observed and fitted activity traces seem to match; however, closer inspection of the residuals reveals that they are neither Gaussian nor white. For a sufficient model fit, residuals should form a sequence of independently distributed Gaussian variables. B–C, Residual analysis shows the linear rate model is an inadequate model. The histogram of residuals is shown for the PD neuron (B). Lilliefors' procedure rejected the null hypothesis that the residuals are samples of a Gaussian distribution (red line; ). Furthermore, residuals should be uncorrelated over consecutive time bins. A scatter plot of the residuals for two consecutive time bins (C) illustrates that residuals are not independent of each other (histogram-based test of serial independence, , , using 10 bins per dimension). Here, residuals are normalized by their empirical cumulative density function. Thus, fundamental assumptions of the linear model, i.e., that the difference between the observed signal and the model is Gaussian and random in time, are violated. (TIF) [file pcbi.1003138.s003.tif]

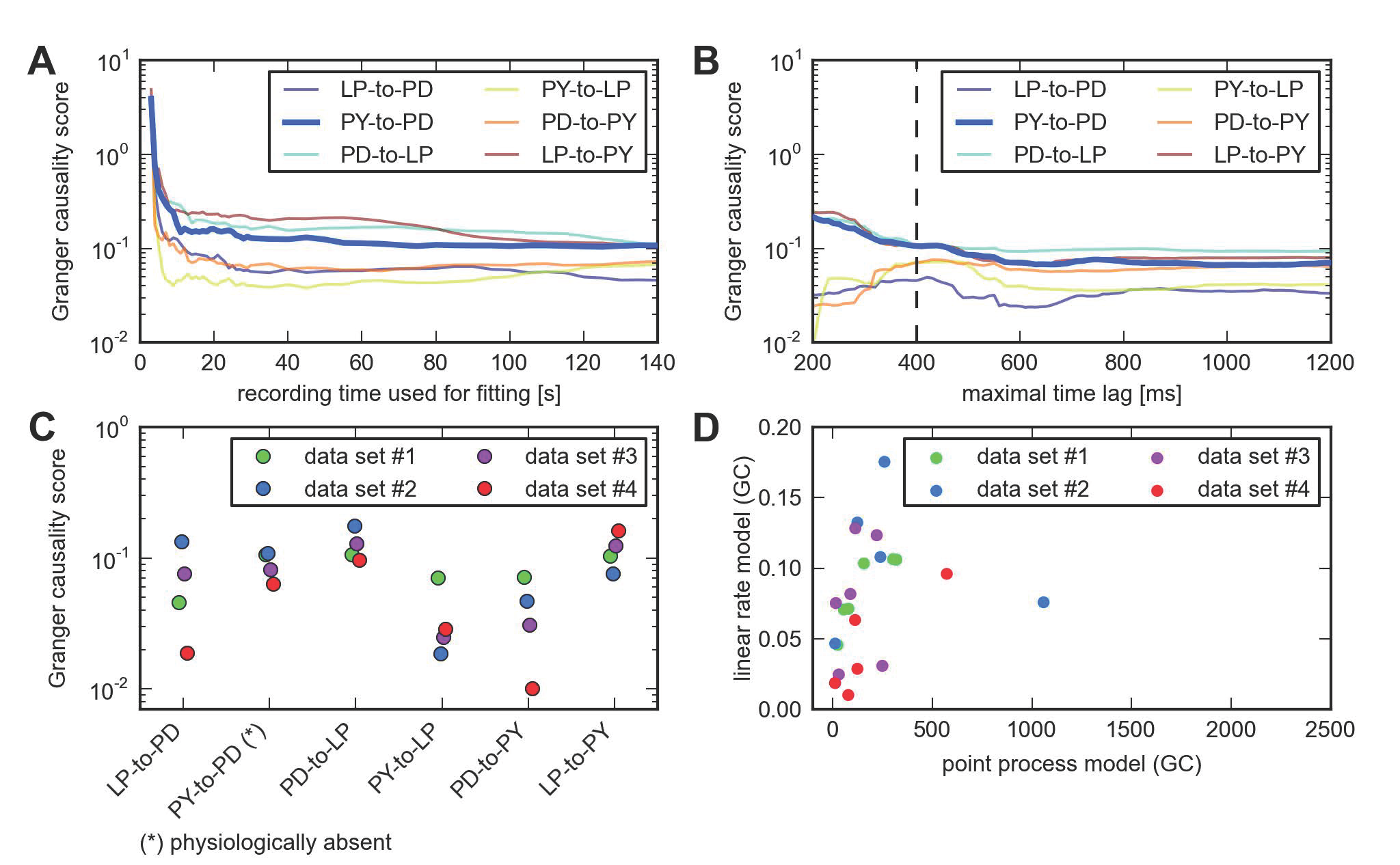

Supplement: Figure S4 — A linear rate model using the Granger causality criterion to define coupling does not accurately reproduce the known physiological connectivity for a wide range of parameter choices. A, Results of linear Granger causality analysis for varying amounts of data used for fitting. For comparison, previous analysis [29] used 5 s. The physiologically absent connection (thick blue line) is never among the weakest connections. B, Results of linear Granger causality analysis for varying maximal time lags of the auto- and cross-regressive filters. Previous analysis [29] used 400 ms (vertical dashed line). The physiologically absent connection (thick blue line) is never among the weakest connections. All connections are highly statistically significant regardless of the maximal time lag. C, Linear Granger causality fails to recover the known physiological connectivity for all four data sets. Horizontal scatter is for visualization only. The GC scores are not the same as in [29] because we used the full set of recordings. Relative magnitudes are consistent with previous analysis of [29]. Both analyses used a multivariate version of the model. D, Granger causality scores for the nonlinear point process model (horizontal axis) and the linear rate model (vertical axis) are correlated (, ). The scatter plot shows the six cross-couplings for each of the four data sets. (TIF) [file pcbi.1003138.s004.tif]
