# Supplementary material for: Successful Reconstruction of a Physiological Circuit with Known Connectivity from Spiking Activity Alone
Source: PLoS Comput Biol. 2013 Jul 11;9(7):e1003138. doi: 10.1371/journal.pcbi.1003138 (PMC3708849; doi:10.1371/journal.pcbi.1003138)
Supplement: Text S1 — Additional methods. We present detailed information about methods on evaluating goodness-of-fit for point process models and linear rate models as well as how to quantify the uncertainty in the coupling strength estimates. (PDF) [file pcbi.1003138.s005.pdf]

Successful reconstruction of a physiological circuit with known connectivity from spiking activity alone. - Text S1

Felipe Gerhard[1,*], Tilman Kispersky[2], Gabrielle J Gutierrez[2,3], Eve Marder[2], Mark Kramer[4], Uri Eden[4]
1 Brain Mind Institute, Ecole Polytechnique Fédérale de Lausanne (EPFL), Lausanne, Switzerland
2 Biology Department and Volen Center, Brandeis University, Waltham, MA
3 Group for Neural Theory, École Normale Supérieure, Paris, France
4 Department of Mathematics and Statistics, Boston University, Boston, MA
∗ E-mail: felipe.gerhard at epfl.ch

Goodness-of-fit of multivariate point process models

Goodness-of-fit tests serve to identify possible model misspecifications. An extensive battery of goodness-of-fit tests specifically tailored to point process models were performed using the multivariate time-rescaling theorem [1] in conjunction with the extension of the time-rescaling test for time-discrete models [2,3]. The multivariate time-rescaling procedure is an extension of the classical change-of-time theorem [4] to sets of coupled point process models (e.g., simultaneously recorded spike trains). Briefly, each observed spike train is rescaled according to its estimated conditional intensity. After rescaling, the processes should be Poisson processes with unit rate. Deviance from the Poisson process assumption is commonly assessed with a Kolmogorov-Smirnov (KS) plot, in which the difference between the empirical and theoretical cumulative density function (CDF) of the inter-spike interval distribution is plotted against the empirical CDF. For a Poisson process, the theoretical inter-spike interval distribution is given by the exponential distribution. The result of the KS can be visually assessed by checking whether the residual, defined as the difference between observed and theoretical CDF, fully lies within the analytical 95% confidence bounds. If so, the goodness-of-fit test is passed.

If, and only if, the dependencies between the spike trains have been properly accounted for by the model, the rescaled processes will also be statistically independent of each other. This is the basis of the multivariate test procedure [1]. One consequence of independent Poisson processes is their superposition is a Poisson process with homogeneous rate as well. Hence, we also test for the independence of the individual processes by super-imposing all processes and performing a KS test on the inter-spike intervals of the superimposed spike train.

The exponential inter-spike interval distribution is only a necessary condition for a Poisson process. An additional necessary condition is that intervals must be statistically independent of each other. We assess the independence of consecutive intervals by plotting pairs of consecutive normalized ISIs. In case of independence, points should uniformly fill the unit area. We formally test this by creating a two-dimensional histogram with 10 bins per dimension and assess independence by a standard χ^2 test of independence.

Furthermore, if we identify each spike in the superimposed spike train by its neuron identity, we obtain the mark sequence, defined as the sequence of integer numbers $(1, ..., N)$ for N neurons. Independence of the individual rescaled processes implies an independent mark sequence. Specifically, we test the independence of consecutive marks by crosstabulating them and performing another χ^2 test of independence, following the test procedure proposed in [1].

The multivariate goodness-of-fit test is passed if the residuals of all neurons and of their superposition fully lie within the confidence bounds and the χ^2 tests does not reject the independence assumptions ($P > 0.05$). These multivariate goodness-of-fit tools allow for an easy visual and formal assessment of how well the dependency structure between the simultaneously recorded spike trains is captured by the couplings in the model.

Goodness-of-fit of the linear rate models

Inspection of the residual structure of the model often provides insights into what aspects of the data are misidentified. Residuals are given by the time series $\tilde{\epsilon}(t)$ (see Equation (8) of the main manuscript). Particularly, $\tilde{\epsilon}(t)$ should form an i.i.d. sequence of normally distributed variables. The normality assumption was assessed using Lilliefors' procedure, which tests the null hypothesis that the samples come from a normal distribution with unknown mean and variance. Furthermore, residuals should be independent of each other. Independence of the whole sequence of residuals includes independence of consecutive residuals. Any temporal structure of the residuals generally points to a misspecified model. To check this particular assumption of the model, residuals were first normalized using their empirical cumulative density function to obtain marginally uniformly distributed variables. Then, independence can be visually assessed by plotting $\tilde{\epsilon}(t_{i+1})$ versus $\tilde{\epsilon}(t_i)$: For independent variables, the points should uniformly fill the unit area. We quantitatively tested for independence of consecutive intervals by constructing a 2D histogram (10 bins per dimension) and applying a χ^2 test of independence. A more rigorous goodness-of-fit procedure would extend the test for independence for pairs of residuals that are not immediate neighbors.

Variability of coupling strengths

To measure variability of coupling strengths, we first computed the covariance structure of the parameter estimates: $cov(\hat{\beta}) = I^{-1}(\hat{\beta})$ where $I(\hat{\beta})$ is the observed Fisher information matrix at the maximum likelihood (ML) estimate [5] and then used Gaussian error propagation for correlated variables with Equation (9) of the main manuscript. We obtain:

$$\sigma^2_{CS(X\to Y)} = \sum_{i,j}^{m_c} D_i^* D_j^* cov(\hat{\beta})_{ij}. \tag{1}$$

with $D_i^* = \int_0^\infty D_i(\tau)d\tau$ being the integral of the ith basis function of the interaction filter. We numerically verified Equation (1) with resampling 10,000 parameter samples from the distribution $N(\hat{\beta}, I^{-1}(\hat{\beta}))$, calculating the coupling strength $CS(X \to Y)$ and the standard deviation of the resulting distribution $\sigma_{CS(X\to Y)}$.

References

1. Gerhard F, Haslinger R, Pipa G (2011) Applying the Multivariate Time-Rescaling Theorem to Neural Population Models. Neural Computation 23: 1452–1483.

2. Haslinger R, Pipa G, Brown E (2010) Discrete Time Rescaling Theorem: Determining Goodness of Fit for Discrete Time Statistical Models of Neural Spiking. Neural Computation 22: 2477–2506.

3. Gerhard F, Gerstner W (2010) Rescaling, thinning or complementing? On goodness-of-fit procedures for point process models and Generalized Linear Models. In: Lafferty J, Williams CKI, Shawe-Taylor J, Zemel RS, Culotta A, editors, Advances in Neural Information Processing Systems 23. Curran Associates, Inc., pp. 703–711.

4. Brown EN, Barbieri R, Ventura V, Kass RE, Frank LM (2002) The Time-Rescaling Theorem and Its Application to Neural Spike Train Data Analysis. Neural Computation 14: 325–346.

5. Pawitan Y (2001) In all likelihood: statistical modelling and inference using likelihood. Oxford: Oxford University Press.
